# Spinal Astrocytic Activation Contributes to Mechanical Allodynia in a Rat Chemotherapy-Induced Neuropathic Pain Model

**DOI:** 10.1371/journal.pone.0060733

**Published:** 2013-04-09

**Authors:** Xi-Tuan Ji, Nian-Song Qian, Tao Zhang, Jin-Mao Li, Xin-Kui Li, Peng Wang, Dong-Sheng Zhao, Gang Huang, Lei Zhang, Zhou Fei, Dong Jia, Le Niu

**Affiliations:** 1 Department of Neurosurgery, Xijing Hospital, Fourth Military Medical University, Xi’an, People’s Republic of China; 2 Department of Hepatobiliary Surgery, PLA General Hospital, Beijing, People’s Republic of China; 3 Department of Orthopaedics, Guangzhou General Hospital of Guangzhou Military Command, Guangzhou, People’s Republic of China; 4 Department of General Surgery, Tangdu Hospital, Fourth Military Medical University, Xi’an, People’s Republic of China; 5 Department of Orthopaedics, Xijing Hospital, Fourth Military Medical University, Xi’an, People’s Republic of China; 6 Department of Neurosurgery, Tangdu Hospital, Fourth Military Medical University, Xi’an, People’s Republic of China; 7 School of Basic Medical Sciences, Fourth Military Medical University, Xi’an, People’s Republic of China; University of Pecs Medical School, Hungary

## Abstract

Chemotherapy-induced neuropathic pain (CNP) is the major dose-limiting factor in cancer chemotherapy. However, the neural mechanisms underlying CNP remain enigmatic. Accumulating evidence implicates the involvement of spinal glia in some neuropathic pain models. In this study, using a vincristine-evoked CNP rat model with obvious mechanical allodynia, we found that spinal astrocyte rather than microglia was dramatically activated. The mechanical allodynia was dose-dependently attenuated by intrathecal administratration of L-α-aminoadipate (astrocytic specific inhibitor); whereas minocycline (microglial specific inhibitor) had no such effect, indicating that spinal astrocytic activation contributes to allodynia in CNP rat. Furthermore, oxidative stress mediated the development of spinal astrocytic activation, and activated astrocytes dramatically increased interleukin-1β expression which induced N-methyl-D-aspartic acid receptor (NMDAR) phosphorylation in spinal neurons to strengthen pain transmission. Taken together, our findings suggest that spinal activated astrocytes may be a crucial component of the pathophysiology of CNP and “Astrocyte-Cytokine-NMDAR-neuron” pathway may be one detailed neural mechanisms underlying CNP. Thus, inhibiting spinal astrocytic activation may represent a novel therapeutic strategy for treating CNP.

## Introduction

Chemotherapy-induced neuropathic pain (CNP) is a common, dose-limiting side effect of cancer chemotherapeutic agents which include the vinca alkaloids such as vincristine. Similar to other types of neuropathic pain, CNP is manifested as allodynia, that is, when normally nonpainful stimuli become painful, and hyperalgesia, an increased sensitivity to normally painful stimuli [1]. CNP limits the duration of treatment and impairs the quality of life [2]. So far, there have been no validated measures for the prevention or treatment of CNP with ambiguous underlying mechanisms. At this point, it might be of critical importance to address the mechanisms of CNP and consequently obtain effective treatment strategies for this devastating disease [3].

Unlike neuropathic pain induced by trauma and diabetes, pain in paclitaxel-treated and vincristine-treated rats occurs in the absence of axonal degeneration in peripheral nerves [4], [5], suggesting that the mechanisms underlying CNP are elusive and complex. It is hypothesized that the fundamental pathology for CNP is the toxic effect on axonal mitochondria. Impaired mitochondrial function may lead to membrane depolarization and action potentials [6]. In neurophysiological studies, hypersensitivity of C-fiber nociceptors has been noted following the treatment with the anti-tumor agent vincristine [7]. As well, central sensitization of spinal wide dynamic range neurons has also been observed with vincristine treatment [8]. However, the exact underlying mechanisms of CNP remain unidentified.

According to classic pain research, the pain related pathway has been described simply as a serial chain of neuronal elements [9]–[11]. However, recent lines of evidence suggest that spinal glia (including astrocytes and microglia) also play important roles in the initiation and maintenance of neuropathic pain, and inhibiting spinal glial cell activation may be a potential strategy to alleviate neuropathic pain [12], [13]. Amongst the inhibitors of glial cells, minocycline, a semisynthetic second-generation tetracycline, has emerged as a selective inhibitor for microglial activation and proliferation [14], [15]. It has been noted that intraperitoneal or intrathecal administration of minocycline exerted anti-allodynic and anti-hyperalgesic effects on spinal nerve ligation induced neuropathic pain [16], [17], sciatic nerve constriction induced neuropathic pain [18], peripheral inflammation induced pain [19] and spinal nerve transection induced neuropathic pain [20]. On the other hand, L-α-aminoadipate (LAA) is used to inhibit astrocytes due to its hallmark as a specific astrocytic toxin [21]. In recent studies, it has been demonstrated that intrathecal administration of LAA blocked the activation of spinal astrocytes and exerted analgesic effect on spinal nerve ligation induced neuropathic pain [22] and chronic pancreatitis induced abdominal pain [23].

With regard to the relation between spinal glia and CNP, two recent studies showed that spinal microglia was not activated in rat models of CNP [24], [25]. Zheng et al. (2011) showed that there was no microglial activation in the rats treated with paclitaxel, vincristine, oxaliplatin, or 2′,3′-dideoxycytidine, whereas the state of spinal astrocyte in these CNP rats was not evaluated [24]. Zhang et al. (2012) observed both spinal astrocyte and microglia in paclitaxel induced CNP rat model, and showed that paclitaxel treatment induced a rapid and persistent activation of spinal astrocytes but not the activation of microglia. Zhang’s research group used minocycline to prevent activation of astrocytes without performing behavioural test to evaluate the analgesic effects of minocycline [25].

It is noteworthy that the introduction of a vincristine-induced CNP rat model might facilitate the studies of the neural mechanisms underlying CNP. In the model, rats are surgically implanted with mini-osmotic pumps set to continuously deliver vincristine sulfate [26], [27]. Recent studies indicated that this rat model could ideally mimic the chronic pain states that occur in CNP patients [28]. In the present study, we investigated the role of spinal glia in the pathophysiology of CNP by using this CNP model. LAA and minocycline were used to inactivate astrocyte and microglia, respectively, to identify the roles of spinal glial cells in the development of CNP. In addition, intrathecal treatment with a scavenger of reactive oxygen species (ROS) was performed to test whether oxidative stress mediates the development of glial activation. The role of inflammatory cytokine on NMDAR activation was also investigated.

## Materials and Methods

### Animals

Adult male Sprague–Dawley rats, weighing 200 g, were used. Rats were housed under standard conditions (temperature 22±2°C, 12 h-light/dark cycle) with food and water available ad libitum. All procedures of our experiments were approved by the Committee of Animal Use for Research and Education of the Fourth Military Medical University (Xi’an, PR China), and all efforts were made to minimize the number of animals used and their suffering, in accordance with the ethical guidelines for animal research [29]. (Permit Number: fmmu-12-6888).

### Mini-osmotic Pump Implantation

As described previously [26], [27], rats were anesthetized with halothane (5% to induce, 2–3% to maintain), and their right external jugular vein was catheterized (PE60 tubing) with a vincristine-filled miniosmotic pump (0.5 µl/h, 14 days; Alzet Model 2002, Durect Corporation, Cupertino, CA, USA) that had been primed overnight to deliver 30 mg/kg/day vincristine sulfate (Sigma-Aldrich, St Louis, MO, USA) (Vincristine group). Control rats were implanted with primed, mini-osmotic pumps containing the 0.9% saline vehicle (Sham group).

### Antibodies

Primary antibodies: mouse anti-GFAP IgG (astrocytic marker; Chemicon, Temecula, CA, USA), mouse anti-NeuN IgG (neuronal marker; Chemicon), mouse anti-OX42 IgG (microglial marker; Chemicon), rabbit anti-IL-1β IgG (Endogen, Rockford, IL, USA), rabbit anti-P-ser896 NR1 IgG (Millipore, Bedford, MA, USA), and goat anti-IL-1RI IgG (R&D Systems; Minneapolis, MN, USA). Secondary antibodies: FITC-labeled donkey anti-mouse IgG (Chemicon), Cy3-labeled donkey anti-rabbit IgG (Chemicon), FITC-labeled donkey anti-goat IgG (Chemicon).

### Drugs

Chemicals and their sources were as follows: L-α-aminoadipate (LAA, astrocytic specific inhibitor; Sigma, St. Louis, MO, USA), minocycline (microglial specific inhibitor; Sigma), phenyl N-tert-butylnitrone (PBN, scavenger for reactive oxygen species; Sigma, St. Louis, MO, USA), pentoxifylline (cytokine inhibitor; Polfilin, Polfarma, Poland), interleukin-1 receptor antagonist (IL-1ra; Amgen, Thousand Oaks, CA, USA), 5-aminophosphonovaleric acid (AP5, NMDA receptor antagonist; Sigma) and (5*R*,10*S*)-(++)-5-methyl-10,11-dihydro-5*H*-dibenzo[*a,d*]cyclo-hepten-5,10-imine hydrogen maleate (MK-801, non-competitive NMDA receptor antagonist; Sigma).

### Experimental Design

In the first series of experiments, rats were divided into Naive group, Sham group, Vincristine group. Pain behavior, immunostaining of GFAP or OX42, and Western blot of GFAP, OX42, IL-1β or P-NR1 were detected once per day from 1 day post osmotic pump implantation (1 d) to 35 days post osmotic pump implantation (35 d) (n = 10/group). Body weight, motor function, and blood plasma levels of aspartate aminotransferase (AST) and alanine aminotransferase (ALT) were detected once per week from 1 week post osmotic pump implantation (1 w) to 5 weeks post osmotic pump implantation (5 w) to monitor the toxicity of vincristine (n = 10/group).

In the second series of experiments, rats of Vincristine group were injected intraperitoneally with PBN (1.5 mg/kg/day) from 1 d to 7 d, and spinal cords were harvested for Western blot analysis of GFAP or OX42 at 7 d (n = 10).

In the third series of experiments, saline, LAA, minocycline, pentoxifylline, IL-1ra, AP5 or MK-801 was injected intrathecally in Vincristine group at 1 w. After injection, pain behavior was immediately measured (n = 10).

In the fourth series of experiments, LAA was injected intrathecally in Vincristine group at 1 w, and one hour later spinal cords were harvested for Western blot analysis of IL-1β (n = 10). Double-labeling immunofluorescence of IL-1β with GFAP, OX42 or NeuN was performed in the L5 spinal cord sections.

In the fifth series of experiments, LAA, pentoxifylline or IL-1ra was injected intrathecally in Vincristine group at 1 w, and one hour later the spinal cords were harvested for Western blot analysis of P-NR1 (n = 10). Double-labeling immunofluorescence of IL-1RI and P-NR1 was performed in the L5 spinal cord sections.

### Rotarod Test

In order to assess whether the dosage of vincristine used in the present experiment induced impairment of motor function, which might influence the pain behavioral results, the motor function of vincristine treated rats was assessed by the rotarod test [30]. Rats were placed on the Ugo Basile 7650 Rotarod accelerator treadmill (Ugo Basile, Varese, Italy) set at a constant speed of 25 RPM. As the animal took a grip of the drum, the accelerator mode was selected on the treadmill, the rotation rate of the drum was increased linearly. Thereafter, the time was measured from the start of the acceleration period until the rat fell off the drum. The cut-off time was 30 s.

### Hepatotoxicity Determinations

The blood from tail vein was centrifuged at 1000×*g* for 5 min, and the plasma was collected. Plasma levels of aspartate aminotransferase (AST) and alanine aminotransferase (ALT) were determined with commercial test kits (Sigma) as indexes of liver injury.

### Intrathecal Catheter Insertion and Drug Administration

A polyethylene-10 catheter (Becton-Dickinson, Sparks, MD, USA) was intrathecally inserted according to a previous method [31]. The rats were allowed to recover for 1 day. Only the rats judged as neurologically normal were used for the subsequent drug administration. The dosage of each drug used in the present study was according to the previous reports. Treatment group received intrathecal injection of LAA (50, 100, 150 nmol) [23], minocycline (100 µg) [32], pentoxifylline (150 nmol) [33], IL-1ra (100 µg) [34], AP5 (40 pmol) [35] or MK-801 (100 pmol) [35], while the same volume of normal saline was injected in control group. Each drug was dissolved in 10 µl of saline and injected intrathecally by the way of a single acute administration.

### Pain Behavioral Test

The rats were adapted to the testing situation for at least 15 min before stimulation was initiated. The observers of the behaviors were blinded to the treatment of the rats. To observe how different drug treatments affected the pain threshold, behavioral tests were performed 12 h before the drugs administration to provide baseline scores. After intrathecal drug injection, pain threshold was measured for every 10 min.

#### Von Frey monofilaments

As previously described [36], a set of von Frey monofilaments (Stoelting, Chicago, IL, USA) was used to test the mechanical withdrawal threshold of the hindpaws. The monofilaments were applied with increasing force until the rat withdrew the paw. Lifting of the paw due to normal locomotory behavior was ignored. Each monofilament was applied five times. The threshold was taken as the lowest force that evoked a brisk withdrawal response to one of the five repetitive stimuli. The withdrawal thresholds were measured three or four times in order to obtain two consecutive values that differed not more than 10%.

#### Hargreaves’ method

Thermal nociceptive threshold to radiant heat was quantified using the paw withdrawal test [37]. Briefly, rats were placed in a Perspex enclosure, without restraint and a movable infrared radiant heat source placed directly under the plantar surface of the hind paw (Ugo Basile, Como, Italy). The paw withdrawal latency to radiant heat was defined as the time from onset of the radiant heat to the withdrawal of the rat hind paw. The average of three estimations was taken to yield a mean paw withdrawal latency.

### Immunofluorescence Histochemical Staining

#### Tissue preparation

Rats were anesthetized and perfused transcardially with paraformaldehyde. The L5 spinal cord were removed and transferred into 30% sucrose in 0.1 M phosphate buffer (PB, pH 7.4) for cryoprotection. Spinal cord was cut into 30 µm thick sections and collected in 0.01 M phosphate-buffered saline (PBS, pH 7.4).

#### Single immunofluorescence

After washed in PBS, the spinal cord sections were incubated sequentially with: (1) mouse anti-GFAP IgG (1∶500) or mouse anti-OX42 IgG (1∶200) in 0.0l M PBS containing 5% (v/v) normal donkey serum (NDS), 0.3% (v/v) Triton X-100, 0.05% (w/v) NaN_3_ and 0.25% (w/v) carrageenan (PBS-NDS, pH 7.4) for 48 h at 4°C; (2) FITC-labeled donkey anti-mouse IgG (1∶200) in PBS-NDS for 12 h at 4°C.

#### Double immunofluorescence

After washed in PBS, the sections were incubated sequentially with: (1) rabbit anti-IL-1β IgG (1∶300) was double labelled with mouse anti-GFAP IgG (1∶500), mouse anti-NeuN IgG (1∶1000) or mouse anti-OX42 IgG (1∶200) in PBS-NDS for 48 h at 4°C; (2) a mixture of FITC-labeled donkey anti-mouse IgG (1∶200) with cyanin 3 (Cy3)-labeled donkey anti-rabbit IgG (1∶200) in PBS-NDS for 12 h at 4°C.

Also, rabbit anti-P-ser896 NR1 IgG (1∶500) was double labelled with goat anti-IL-1RI IgG (1∶600). The second antibodies were Cy3-labeled donkey anti-rabbit IgG (1∶200) and FITC-labeled donkey anti-goat IgG (1∶200).

Between each step, the sections were washed with PBS for three times. After staining, the sections were coverslipped with a mixture of 50% (v/v) glycerin and 2.5% (w/v) triethylene diamine (anti-fading agent) in PBS, and observed with a confocal laser scanning microscope (Olympus FV1000, Tokyo, Japan) under appropriate filters for green-emitting FITC (excitation 490 nm; emission 520 nm) and for red-emitting Cy3 (excitation 552 nm; emission 565 nm).

### Western Blot Analysis

Rats were anesthetized and the spinal cord innervated by the L4–L5 dorsal roots were rapidly removed. The collected tissue was mechanically homogenized and centrifuged. The supernatant was collected and stored at –80°C. Protein concentrations of the supernatant were determined using the BCA Protein Assay Kit (Pierce, Rockford, IL, USA). Proteins of interest were separated by SDS-PAGE electrophoresis (20 µg of total protein per well), and transferred onto nitrocellulose membranes. The membranes were placed in a blocking solution (Tris-buffered saline with 0.02% Tween and 5% non-fat dry milk powder) for 1 h, and incubated overnight with mouse anti-GFAP IgG (1∶500), mouse anti-OX42 IgG (1∶200), rabbit anti-IL-1β IgG (1∶300) or rabbit anti-P-ser896 NR1 IgG (1∶500). After washing, the membranes were incubated in peroxidase-conjugated secondary antibody (1∶1000; Santa Cruz) for 1 h, and then the membranes were detected by the enhanced chemiluminescence detection method (Amersham Pharmacia Biotech Inc., Piscataway, NJ, USA). The densities of protein blots were analyzed by using Labworks Software (Ultra-Violet Products Ltd., Cambridge, UK) and normalized to β-actin levels.

### Data Analysis

All data were collected by experimenters blinded to the surgery and reagents treatments and statistical analyses were done by using SPSS software (version 12). Data were expressed as mean ±standard error mean (mean±SEM). Statistical analysis of the data was carried out with a one-way analysis of variance (ANOVA) followed by Bonferroni post hoc analysis. Comparisons between two means were performed by a Student’s T-test. Significance level was set at *P*<0.05.

## Results

### Mechanical Allodynia Occurred in Vincristine Treated Rats

**Figure 1:**
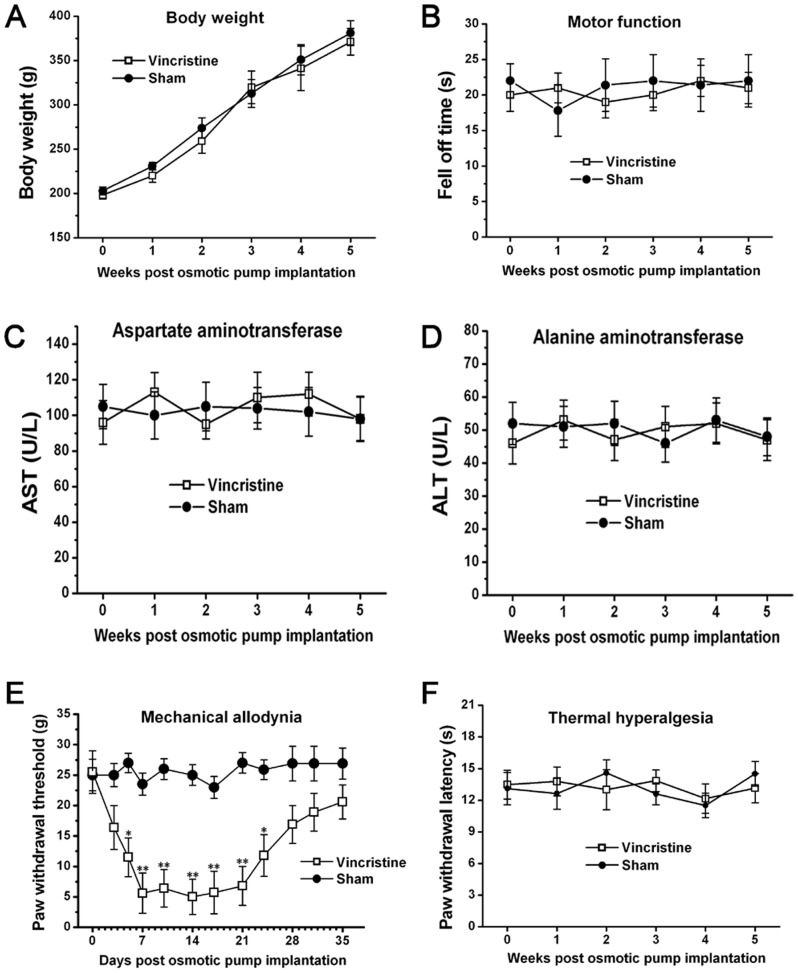
Mechanical allodynia occurred in vincristine treated rats. With regard to (A) body weight, (B) motor function and plasma levels of (C) aspartate aminotransferase and (D) alanine aminotransferase, there was no difference between Sham group and Vincristine group. (E) Compared with Sham group, the paw withdrawal threshold of Vincristine group was significantly decreased. (F) No significant changes in thermal withdrawal thresholds were observed between Sham group and Vincristine group. All data were calculated as mean ± SEM (n = 10/group/week). *P<0.05, **P<0.01 vs. Sham group in E.

No difference in body weight was observed between Sham group (381±14.1 g; 5 w) and Vincristine group (371.3±15.1 g; 5 w), and the body weight of these two groups was persistently elevated through the period tested (Fig. 1A).

Also, no difference in motor function and plasma levels of aspartate aminotransferase and alanine aminotransferase was observed between Sham group and Vincristine group, and these values maintained at normal level from 1 w to 5 w (Fig. 1B–D).

Compared to Sham group (27±2.6 g; 5 d), the paw withdrawal threshold of Vincristine group significantly decreased at 5 d (11.5±3.2 g), reached the lowest value at 1 w (5.6±1.3 g), and thereafter maintained at low level till 3 w (6.8±2.2 g) (n = 10/group; *P*<0.05). After 3 w, the paw withdrawal threshold of Vincristine group gradually increased to the basal level (20.6±2.8 g; 5 w) (Fig. 1E).

In contrast with the severe mechanical allodynia, no significant changes in thermal withdrawal thresholds were observed between Sham group (14.5±1.2 s; 5 w) and Vincristine group (13.2±1.4 s; 5 w) (Fig. 1F).

Thus, these data indicated that vincristine treatment induced mechanical allodynia and exerted no significant toxicity.

### Mechanical Allodynia was Induced by Spinal Astrocytic Activation, and Chemotherapy-induced Oxidative Stress Might be One of the Causative Factors for Astrocytic Activation

**Figure 2:**
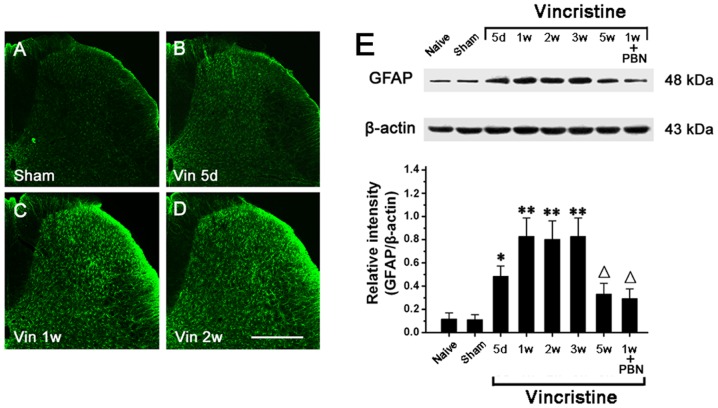
Spinal astrocyte was activated in vincristine treated rats. (A–D) Compared to Sham group, GFAP-like immunoreactivity in spinal dorsal horn of Vincristine group was significantly increased. Bar = 200 µm. (E) Compared to Naive group and Sham group, Western blot analysis showed that spinal GFAP expression was significantly increased in Vincristine group. A systemic treatment with PBN (scavenger for reactive oxygen species) significantly reduced GFAP overexpression in Vincristine group at 1 week post osmotic pump implantation (1 w). All data were calculated as mean ± SEM (n = 10/group/week). *P<0.05, **P<0.01 vs. Naive group and Sham group; △P<0.05 vs. Vincristine group at 1 w. Vin: vincristine; d: day; w: week; GFAP: astrocytic marker; PBN: phenyl N-tert-butylnitrone.

Immunohistochemistry indicated that compared to Sham group, GFAP staining was significantly increased in the spinal cord of Vincristine group. Staining of GFAP appeared to be enhanced throughout the spinal dorsal horn, Activated astrocytes had hypertrophied cell bodies and thickened processes with enhanced GFAP-immunoreactivity (Fig. 2A–D).

Using Western blot, we detected that compared to naive group (0.12±0.05; 5 d) and Sham group (0.1±0.03; 5 d), GFAP expression was significantly increased in Vincristine group at 5 d (0.48±0.09). GFAP upregulation peaked at 1 w (0.83±0.16) and thereafter persisted at high level till 3 w (0.84±0.18) (n = 10/group; *P*<0.05). After 3 w, GFAP expression gradually decreased to the basal level (0.32±0.08; 5 w) (Fig. 2E). Thus, the time course of GFAP expression was similar to that of mechanical allodynia.

In addition, a systemic treatment with PBN (reactive oxygen species scavenger) significantly reduced GFAP overexpression, which indicated that Vincristine-induced oxidative stress may mediate the development of astrocytic activation in Vincristine group (Fig. 2E).

**Figure 3:**
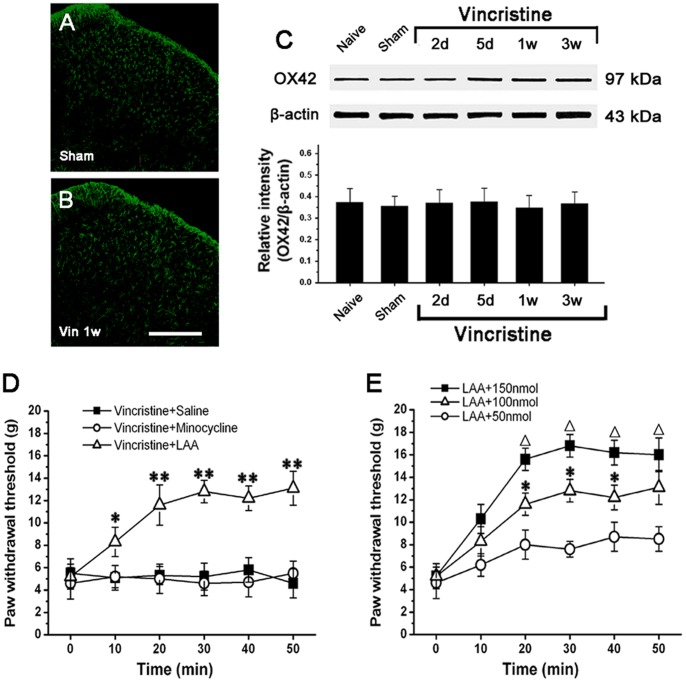
Spinal microglia was not activated in vincristine treated rats, astrocytic specific inhibitor LAA but not microglial specific inhibitor minocycline attenuated mechanical allodynia. (A and B) With regard to OX42-like immunoreactivity in spinal dorsal horn, there was no difference between Sham group and Vincristine group. Bar = 200 µm. (C) No significant difference in OX42 expression in spinal cord was observed among Naive group, Sham group and Vincristine group. In Vincristine group, OX42 expression was unchanged through the period tested. (D) Intrathecal injection of LAA significantly attenuated the allodynia. However, minocycline did not influence the allodynia. (E) Allodynia was attenuated by LAA in a dose-dependent manner. All data were calculated as mean ± SEM (n = 10/group). *P<0.05, **P<0.01 vs. vincristine+Saline group or vincristine+minocycline group in D. *P<0.05 vs. LAA 50 nmol group, △*P*<0.05 vs. LAA 100 nmol group in E. Vin: vincristine; d: day; w: week; min: minute; OX42: microglial marker; LAA: L-α-aminoadipate.

With regard to OX42 expression in spinal cord, immunostaining (Fig. 3A and B) and Western blot (Fig. 3C) showed that there was no difference among naive group, Sham group and Vincristine group at any time points post osmotic pump implantation. In all the rats, OX42 expression was unchanged through the period tested.

We injected LAA or minocycline intrathecally and observed their effects on mechanical allodynia in Vincristine group (1 w). The astrocytic specific toxin LAA (100 nmol) significantly attenuated the allodynia. However, the microglial specific inhibitor minocycline (100 µg) did not influence mechanical allodynia (Fig. 3D).

Intraperitoneal (40 mg/kg; once per day) or intrathecal (100 µg; once per day) repeated administrations of minocycline for eight days from 7 d to 14 d exerted no effects on mechanical allodynia (data not shown).

We further observed that the allodynia was significantly attenuated by LAA in a dose-dependent manner in Vincristine group (1 w) (Fig. 3E). 150 nmol of LAA produced a stronger antiallodynic effect than 100 nmol of LAA (Fig. 3E).

### Spinal Astrocytes Dramatically Increased the Expression of IL-1β which is Related to Mechanical Allodynia

**Figure 4:**
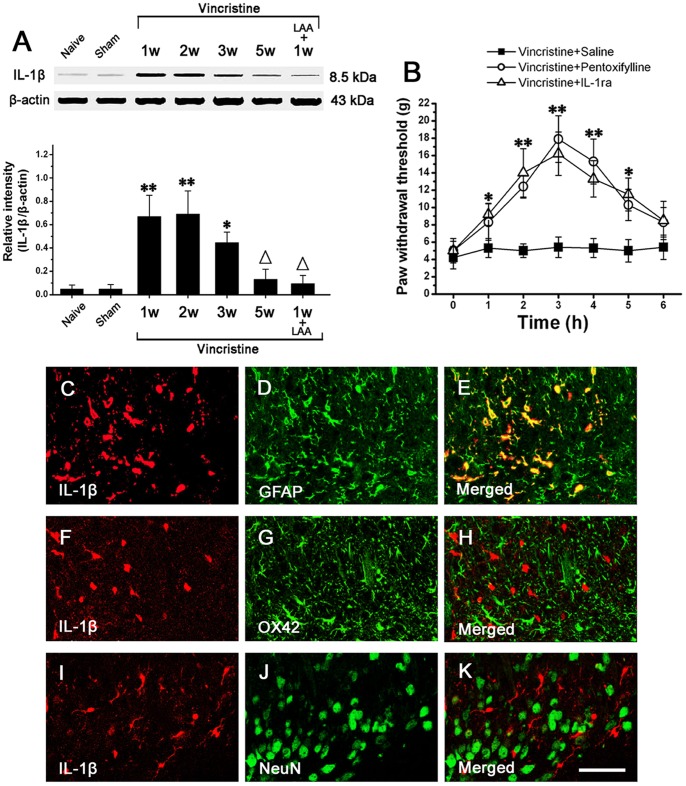
IL-1β overexpression in spinal cord was related to mechanical allodynia in vincristine treated rats, and activated astrocytes were the only source of IL-1β. (A) IL-1β expression was significantly upregulated in spinal cord of Vincristine group compared to Naive group and Sham group. Intrathecal treatment with LAA (astrocytic specific toxin) significantly reduced IL-1β overexpression in Vincristine group. (B) Intrathecal injection of Pentoxifylline (cytokine inhibitor) or IL-1ra (interleukin-1 receptor antagonist) significantly attenuated the allodynia. (C–K) Double immunofluorescent staining showed that IL-1β-immunoreactivity was localized in GFAP-immunopositive cells but not in OX42-immunopositive cells or NeuN-immunopositive cells in spinal cord of Vincristine group. Bar = 20 µm. All data were calculated as mean ± SEM (n = 10/group). *P<0.05, **P<0.01 vs. Naive group and Sham group; △P<0.05 vs. Vincristine group at 1 week post osmotic pump implantation in A. *P<0.05, **P<0.01 vs. vincristine+Saline group in B. IL-1β: interleukin-1β; LAA: L-α-aminoadipate; w: weeks; h: hours.

Western blot analysis showed that compared to Naive group (0.05±0.008; 1 w) and Sham group (0.06±0.01; 1 w), IL-1β expression was significantly increased in Vincristine group. IL-1β upregulation reached a peak at 1 w (0.67±0.16), and then remained at high level till 3 w (0.45±0.11) (n = 10/group; P<0.05). After 3 w, IL-1β expression gradually decreased to the basal level (0.13±0.02; 5 w) (Fig. 4A). Thus, the time course of IL-1β expression was similar to that of GFAP expression. At 1 w in Vincristine group, intrathecally administered LAA significantly down-regulated IL-1β overexpression (Fig. 4A).

At 1 w in Vincristine group, we injected pentoxifylline (cytokine inhibitor) or IL-1ra (interleukin-1 receptor antagonist) intrathecally and observed their effects on mechanical allodynia. Both pentoxifylline and IL-1ra significantly attenuated the allodynia (Fig. 4B).

Subsequent double immunofluorescent staining showed that IL-1β-immunoreactivity was only localized in GFAP-immunopositive cells but not in OX42-immunopositive cells or NeuN-immunopositive cells (Fig. 4C–K).

### IL-1β Induces NMDA Receptor Phosphorylation in Spinal Dorsal Horn Neurons

**Figure 5:**
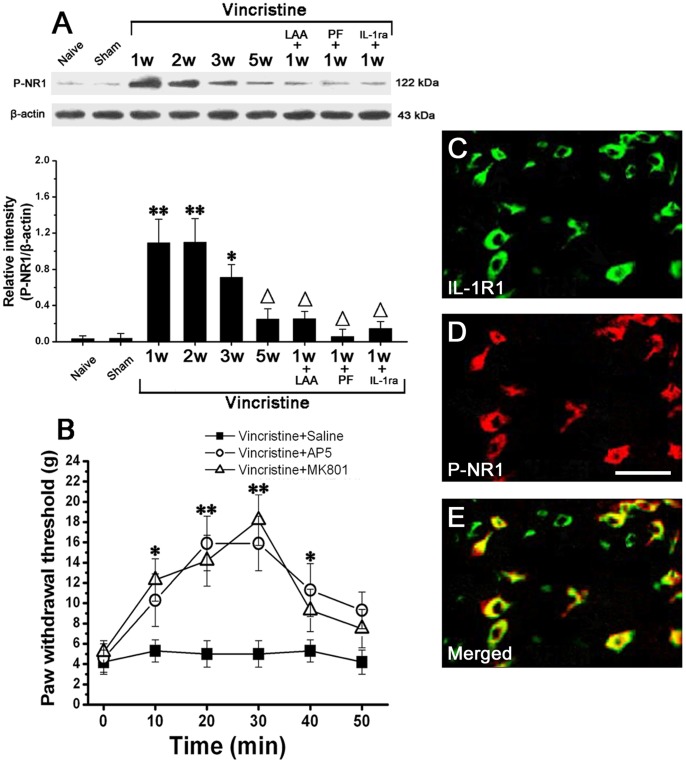
IL-1β released from astrocyte induced NMDA receptor phosphorylation in spinal dorsal horn neurons in vincristine treated rats, which was related to mechanical allodynia. (A) The phosphorylated NR1 subunit of NMDA receptor (P-NR1) was significantly increased in Vincristine group compared to Naive group and Sham group. Intrathecal treatment with LAA (astrocytic specific toxin), PF (cytokine inhibitor) or IL-1ra (interleukin-1 receptor antagonist) significantly reduced P-NR1 in Vincristine group. (B) Intrathecal injection of AP5 (NMDA receptor antagonist) or MK-801 (non-competitive NMDA receptor antagonist) significantly attenuated the allodynia. (C–E) Double immunofluorescent staining showed that P-NR1-immunoreactivity and IL-1RI-immunoreactivity were totally co-localized in spinal dorsal horn of Vincristine group. Bar = 50 µm. All data were calculated as mean ± SEM (n = 10/group). *P<0.05, **P<0.01 vs. Naive group and Sham group; △P<0.05 vs. Vincristine group at 1 week post osmotic pump implantation in A. *P<0.05, **P<0.01 vs. vincristine+Saline group in B. LAA: L-α-aminoadipate; PF: pentoxifylline; IL-1ra: interleukin-1 receptor antagonist; IL-1RI: interleukin-1 receptor1; w: weeks.

Western blot analysis showed that compared to Naive group (0.03±0.007; 1 w) and Sham group (0.04±0.006; 1 w), the phosphorylation of NR1 in Vincristine group was significantly increased, peaked at 1 w (1.1±0.3), and persisted at high level till 3 w (0.7±0.25) (n = 10/group; *P*<0.05). After 3 w, the phosphorylation of NR1 gradually decreased to the basal level (0.21±0.07; 5 w) (Fig. 5A). Thus, the changing course of the level of P-NR1 was similar to that of IL-1β or GFAP. At 1 w in Vincristine group, intrathecally administered LAA, pentoxifylline or IL-1ra significantly reversed the phosphorylation of NR1 (Fig. 5A).

At 1 w in Vincristine group, we injected AP5 (NMDA receptor antagonist) or MK-801 (non-competitive NMDA receptor antagonist) intrathecally and observed their effects on mechanical allodynia. Both AP5 and MK-801 significantly attenuated the allodynia (Fig. 5B).

At 1 w in Vincristine group, double immunofluorescent staining of spinal cord showed that P-NR1-immunoreactivity and IL-1RI-immunoreactivity were totally co-localized (Fig. 5 C–E).

## Discussion

Vinca alkaloids (such as vincristine and vinblastine) have significant efficacy in the treatment of malignant tumors. These agents appear to exert their effects by binding to tubulin and interfering with microtubule dynamics, thus disrupting mitosis [38]. Meanwhile, they also produce sensory abnormalities including severe chemotherapy-induced neuropathic pain (CNP). CNP is characterized as: (i) myalgias; (ii) painful burning paresthesias; (iii) normally nonpainful stimuli become painful (allodynia) and (iv) an increased sensitivity to normally painful stimuli (hyperalgesia) [1]. The hyperalgesia/allodynia may occur with latencies of days to weeks. CNP limits the dosing and duration of treatment and impairs the quality of life [2]. However, CNP is often resistant to standard analgesics, and the mechanisms underlying CNP are not clear [3].

To date none drug or drug class is considered to be both a ‘safe and effective analgesic’ in the treatment of CNP [27], [39]. Tricyclic antidepressants and anticonvulsants are presently recognized as the first-line drugs for the treatment of chemotherapy-induced pain. However, these agents exhibit side-effect profiles (e.g. sedation, dizziness, cardiovascular complications) that, together with incomplete attenuation of pain, constrain therapeutic utility [40], [41]. Opioids, though often prescribed for moderate to severe pain, are sometimes avoided because of their potential for dependence and tolerance, scheduling issues and side effects [42], [43]. The current situation dictates a necessity for unravelling the mechanisms underlying CNP.

A model of vincristine-induced neuropathic pain was developed in rats by using continuous infusion of vincristine for two weeks [26], [27]. This method differs from previous reports in that a continuous infusion technique named mini-osmotic pump was used to provide a consistent blood concentration of vincristine. Implantation of the mini-osmotic pump is a simple procedure with the advantages of performing only once, providing reliable intravenous drug delivery, taking little time to perform, and good tolerance [26].

In this study, we investigated the role of spinal glia in the pathophysiology of CNP by using this CNP rat model. We provided sufficient evidence that spinal astrocytic activation contributed to mechanical allodynia in vincristine treated rats. The neural mechanism in astrocyte induced allodynia may be that spinal activated astrocytes dramatically increased the expression of IL-1β which may induce NMDA receptor phosphorylation in spinal dorsal horn neurons to enhance neuronal activity and pain transmission.

### Spinal Astrocyte Rather than Microglia was Activated in Vincristine Treated Rats, which Contributed to Chemotherapy-induced Neuropathic Pain

CNP is characterized by the development of allodynia, hyperalgesia and spontaneous pain, which are also features of other forms of neuropathic pain. Although it is still unclear how chemotherapeutics interact with the nervous system to induce pain behavioural changes, the neural mechanisms of CNP may be similar to that of other forms of neuropathic pain [3]. Glial cells in central nervous system are now thought to be important regulators of synaptic activity [44].

Recent studies have strongly supported that spinal cord glia (including astrocytes and microglia) and proinflammatory cytokines, such as IL-1β, are involved in the induction and maintenance of neuropathic pain. LAA is a specific astrocytic inhibitor [21] and minocycline is a specific microglial inhibitor [14]. Several reports indicate that intraperitoneal or intrathecal administration of minocycline could relieve neuropathic pain induced by nerve injury [16]–[18], [20] or peripheral inflammation [19]. Also, intrathecal injection of LAA exerted anti-allodynic effects on spinal nerve ligation induced pain [22] and chronic pancreatitis induced pain [23]. These analgesic effects depend on the inhibition of spinal glial activation and proliferation, which consequently lower the expression of proinflammatory cytokines.

To identify which subtype of glial cell (astrocyte or microglia) was involved in CNP, we studied the expression of different glia activation markers in the spinal cord and the influence of different glial inhibitors on mechanical allodynia in vincristine treated rats. We found that GFAP (astrocytic activation marker) rather than OX42 (microglial activation marker) was significantly increased in the spinal cord of vincristine treated rats. LAA (astrocytic specific inhibitor) but not minocycline (microglial specific inhibitor) significantly attenuated the allodynia, which elucidated that astrocytic activation but not microglial activation contributed to allodynia in vincristine treated rats. To the best of our knowledge, this study is the first to use the astrocytic specific inhibitor LAA to attenuate the allodynia in CNP rats.

Similar to our findings, two recent studies showed that there was spinal astrocytic activation but no microglial activation in the rats treated with paclitaxel [24], [25]. However, these two reports did not perform pain behavioural test to evaluate the effect of glial inhibitors (LAA or minocycline). Therefore, there was no way to know if astrocytic activation but not microglial activation contributed to pain in these paclitaxel treated rats. In this study, we found that the mechanical allodynia was dose-dependently attenuated by intrathecal administration of LAA (astrocytic specific inhibitor) in a vincristine induced CNP rat model. Furthermore, we unravelled the molecular mechanism of astrocytic activation which may be the “Oxidative stress- Astrocyte- IL-1β- P-NR1 -neuron” pathway.

Previous studies have clarified that compared with astrocytes, microglial activation contribute more to the initiation of chronic pain. In some neuropathic pain models, microglial activation precedes astrogliosis [45], [46]. In this study, GFAP expression was significantly increased in Vincristine group at 5 d. There was no OX42 overexpression at 2 d and 5 d, indicating that there was no microglial activation prior to astrocytic activation. Moreover, OX42 expression was unchanged through the period tested in Vincristine group. Most importantly, we found that single (intrathecal) or repeated (intrathecal and intraperitoneal) administrations of minocycline did not influence mechanical allodynia, which demonstrated that spinal microglia was really not involved in the initiation and maintenance of chronic pain in Vincristine group.

Our results do not agree with two studies that reported microglial activation with vincristine treatment [47], [48]. Kiguchi et al. (2008) reported microglial activation in the male ICR mice which were intraperitoneally administrated with vincristine in a dose of 0.1 mg/kg once per day for 7 days. Sweitzer et al. (2006) detected a “mild” microglial activation in the male Holtzman rats which were intravenously administrated with vincristine in a dose of 75 µg/kg once per day for 9 days. The reason for the discrepancy is not known. Several variables may be involved, including a rat-mouse species difference, differences in the strain of rats used, and differences in dose and route of the administration of vincristine. Nevertheless, our promising findings regarding LAA-induced alleviation of mechanical allodynia in vincristine treated rats suggest that pharmacological antagonism of astrocytic activation in spinal cord may offer a great advantage in the treatment of CNP.

### Chemotherapy-induced Oxidative Stress Contributed to Spinal Astrocytic Activation in CNP Rat

It has been hypothesized that chemotherapy-evoked neuropathy is a toxic effect on axonal mitochondria [6]. Primary afferent sensory terminals and spinal dorsal horn are the regions of high metabolic demand. It has been shown that the terminals of sensory afferents contain an unusually high concentration of mitochondria [49], [50]. Impaired mitochondrial function produces an excessive amount of reactive oxygen species (ROS), which induces dramatical oxidative stress [51].

ROS generator-induced oxidative stress has been shown to be able to influence the morphology and cell viability of primary cultured astrocytes of rat [52]. Here we hypothesized that chemotherapy-induced oxidative stress may be a key mechanism for the development of spinal astrocytic activation in vincristine treated rats. In the present study, a systemic treatment with PBN (antioxidant for scavenging ROS) significantly reduced GFAP overexpression, which suggests that oxidative stress functions as an initiator of astrocyte activation in vincristine treated rats.

### “Astrocyte-Cytokine-NR1-neuron” Pathway was the Detailed Molecular Mechanism Underlying Astrocytic Activation-induced Allodynia in CNP Rat

Astrocytes under physiological condition do not or only release very low level of neuroexcitatory substances [53]. However, under pathological condition, activated astrocytes release numerous proinflammatory cytokines, amongst which IL-1β has become the research focus [12], [54]. We found that spinal IL-1β expression was significantly increased in vincristine treated rats compared to control rats. Activated astrocytes were the only source of IL-1β release which contributed to mechanical allodynia in vincristine treated rats. In support of our findings, previous studies also reported selective localization of increased IL-1β in spinal astrocytes in bone cancer pain model [55] and Complete Freunds adjuvant-induced inflammatory pain model [56].

Spinal NMDA receptor activation is prominently involved in the induction and maintenance of persistent pain [9], [57]. Recent studies indicate that NMDA receptor activation mainly involves the phosphorylation of its NR1 subunit (increased P-NR1) in the spinal dorsal horn neurons, which is strongly correlated with induction and maintenance of persistent pain [10], [11]. We found that spinal P-NR1 was significantly increased in vincristine treated rats compared to control rats, and P-NR1-immunoreactivity were only localized in spinal dorsal horn neurons. On the other hand, the time course of the upregulation of P-NR1 was similar to that of IL-1β or GFAP. Thus, it was hypothesized that IL-1β released from astrocytes may bind to its endogenous receptor on the neurons to lead to the phosphorylation of NR1 subunit.

Interleukin-1 receptor (IL-1R) is the endogenous receptor for IL-1β. IL-1R contains two subtypes: the type I IL-1R (IL-1RI) and the type II IL-1R(IL-1R II). IL-1RI is a transmembrane molecule that is responsible for IL-1β signaling, whereas IL-1RII lacks an intracellular domain and is incapable of signal transduction [58]. We found that IL-1RI-immunoreactivity were only localized in spinal neurons in vincristine treated rats. In support of our findings, a previous study also reported selective localization of IL-1RI in spinal dorsal horn neurons [59]. Most importantly, double immunofluorescent staining of IL-1RI and P-NR1 in this study strongly supported a close interaction of IL-1β signaling with neuronal NMDA receptor. As expected, subsequent investigations showed that intrathecally administered LAA, pentoxifylline or IL-1ra blocked the phosphorylation of the NR1 in this study. In support of our findings, a recent study showed that phosphorylation of the NR1 was attenuated by LAA in spinal nerve ligation-induced neuropathic pain model [60]. Besides, two previous studies reported that IL-1ra blocked the phosphorylation of the NR1 in inflammatory pain models [34], [61].

Therefore, the above results indicated that spinal activated astrocytes dramatically increased the expression of IL-1β which binds to IL-1RI to induce NMDA receptor phosphorylation in spinal neurons, and neural excitability and pain transmission were finally enhanced.

**Figure 6:**
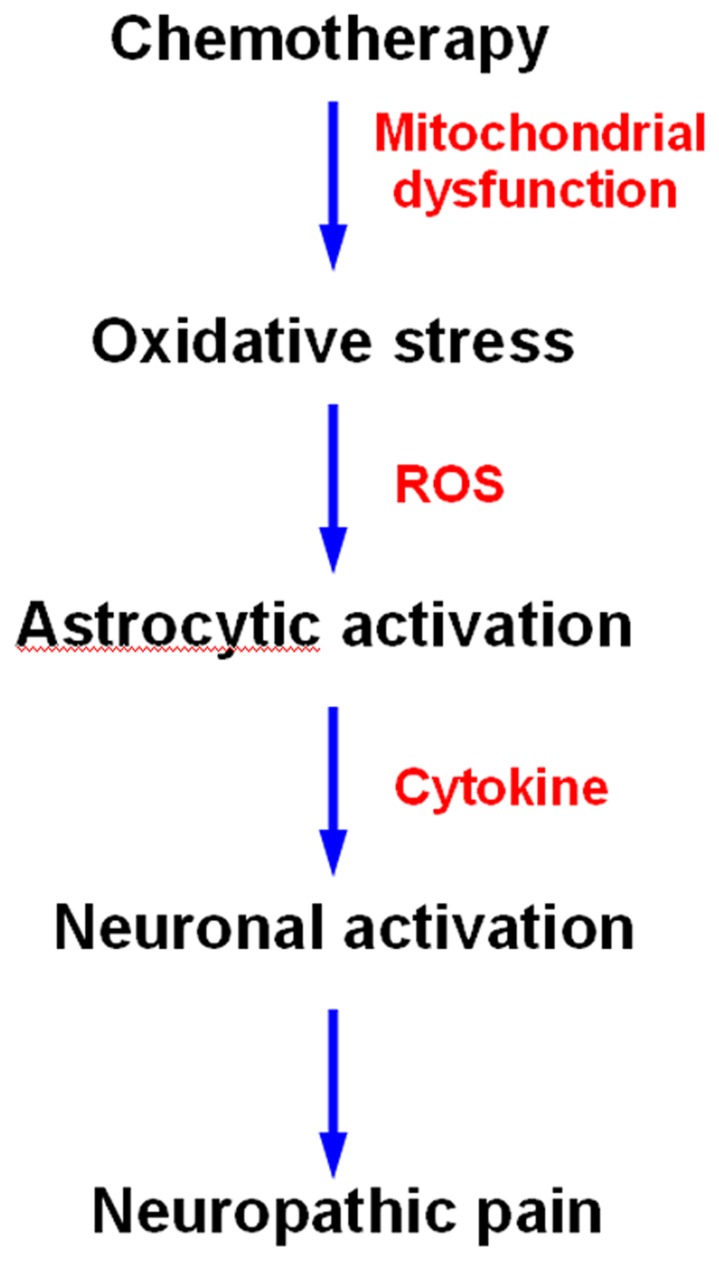
Schematic drawing showed the development of mechanical allodynia in vincristine treated rats. In the situation of chemotherapy, mitochondrial dysfunction may induce oxidative stress which dramatically activates astrocytes in spinal cord. Cytokines like IL-1β are then released from astrocyte and act on spinal dorsal horn neurons to produce chemotherapy-induced neuropathic pain. ROS: reactive oxygen species.

In summary, our study was the first to provide evidence that LAA (astrocytic specific inhibitor) but not minocycline (microglial specific inhibitor) exerted analgesic effects in CNP rat model. Our findings suggested that chemotherapy-induced oxidative stress was the initiator of astrocyte activation, and “Astrocyte-Cytokine-NMDAR-neuron” pathway was the detailed molecular mechanisms underlying astrocytic activation-induced allodynia in CNP rat model (Fig. 6). These findings suggest that spinal astrocytic inhibition may hold a therapeutic promise in the treatment of CNP.
